# Identifying health conditions associated with an increased risk of pancreatic ductal adenocarcinoma at medium term in nationwide electronic health records of primary care physicians

**DOI:** 10.1038/s41416-025-03172-5

**Published:** 2025-08-30

**Authors:** Elie Rassy, Suzette Delaloge, Yannis Slaouti, Thomas Pudlarz, Béranger Lekens, Alice Boilève, Stefan Michiels, Maryam Karimi

**Affiliations:** 1 Department of Cancer Medicine, Gustave Roussy, University Paris-Saclay, Villejuif, France; 2 Department of Biostatistics and Epidemiology, Gustave Roussy, University Paris-Saclay, Villejuif, France; 3 Oncostat U1018, Inserm, University Paris-Saclay, labeled Ligue Contre le Cancer, Villejuif, France; 4 CEGEDIM R&D, Boulogne‐Billancourt, Paris, France; 5 Université Paris Saclay, Orsay, France; 6 Gustave Roussy, INSERM U1279, Villejuif, France

**Keywords:** Cancer epidemiology, Gastrointestinal cancer

## Abstract

**Background:**

Pancreatic ductal adenocarcinoma (PDAC) presents an urgent challenge that necessitates improved early risk identification. We investigated the association between predictors of PDAC present at least two years before diagnosis and PDAC occurence.

**Methods:**

This case-control study used electronic health records from The Health Improvement Network Database (UK). Cases (10,575) were matched with controls (105,750) in a 1:10 ratio by gender, age, follow-up duration, and year of inclusion in the database. Variables included clinical features, comorbidities and blood result abnormalities, reported at least 2 years before PDAC diagnosis or equivalent timeframe for controls. Conditional logistic regression model with backward estimated odds ratios (OR) and 95% confidence interval (CI).

**Results:**

Electronic health records of cases reported higher prevalence of alcohol intake, cigarette smoking, dyslipidemia, increased blood pressure, and diabetes of more than four years’ duration. Independent risk factors included pancreatic cysts (OR = 4.39), pancreatitis (OR = 2.16), dyslipidemia (OR = 1.50), smoking (OR = 1.09), and alcohol intake (OR = 1.09). Laboratory markers associated with higher risk included elevated alkaline phosphatase (OR = 3.21), bilirubin (OR = 2.48), alanine aminotransferase (OR = 1.76), erythrocyte sedimentation rate (OR = 1.27), and decreased albumin (OR = 1.54).

**Conclusions:**

Primary care electronic records can identify individuals at medium-term increased risk of PDAC, thus raises the opportunity to develop early detection models and address modifiable factors.

## Introduction

Pancreatic ductal adenocarcinoma (PDAC) ranks among the deadliest cancers mainly due to its late diagnosis and chemoresistance [1]. In 2022, PDAC accounted for 510,566 new cases and 467,005 cancer-related deaths worldwide [2]. The burden of PDAC has increased considerably over the past decades mainly because of the aging structure of the population together with the increased prevalence of established risk factors [3, 4]. Around 80% of patients with PDAC are diagnosed with locally advanced or metastatic stages that are not amenable to curative surgery [5]. The remaining 20% present with surgically resectable disease that may be cured by combining surgery and chemotherapy [6–9]. Nevertheless and despite extended survivals with multimodal regimens, 50–75% of these patients will relapse within 2 years and ultimately die of their disease [6–8, 10, 11]. Thus, a better understanding of its risk factors and the identification of patients at increased risk of PDAC have become a pressing clinical need [12].

Epidemiological studies on risk prediction models of PDAC have mainly focused on specific high-risk individuals and well established risk factors for PDAC [13–20]. So far, the efforts toward screening for PDAC in the general population were disappointing and did not improve survival outcomes [21]. Subsequently, the latest guidelines restricted screening of PDAC to individuals at high risk of PDAC due to family history or rare inherited pathogenic variants or cystic lesions and did not recommend it in the asymptomatic general population [21–24]. Electronic health records (EHRs) present an interesting analytic opportunity to study risk factors that may be overlooked in traditional studies and to follow the trajectories of these risk factors over many year [25, 26]. To address the challenge of identifying individuals at increased risk of PDAC in the medium term in the general population, we sought to evaluate the comorbidities, clinical data and blood test results before PDAC diagnosis of patients consulting in primary care setting. For this purpose, we analysed a large nested case-control studies drawn from The Health Improvement Network database in the United Kingdom (THIN® UK) to describe the risk factors associated with an increased risk of PDAC at least two years before the PDAC diagnosis.

## Materials and methods

### Data source

The study used data extracted from THIN® Database (A Cegedim Proprietary Database) in UK, which includes various information covering basic demographic characteristics, medical history, medical diagnoses coded according to the International Classification of Disease, 10th Revision (ICD-10), laboratory values, and medication prescriptions (including date, dosage, and duration). THIN® UK was created in 1994 and covers 19 million patients in 2023. Several epidemiologic studies have explored this database to examine patterns of prescription and medical diagnosis [27–35].

THIN® EHR extracts are transmitted by a network of voluntary physicians in compliance with current regulations, including European general data protection regulations. THIN® UK database was granted ethical approval by the NHS South-East Multicentre Research Ethics Committee in 2003 (reference 03/01/073) and was further updated in 2020 by the NHS South Central, Oxford C Research Ethics Committee (reference 20/SC/0011). This retrospective study used secondary deidentified data extract only without mentioning of personal information and as such the legislation did not require additional ethical approval, thus the requirement for obtaining informed consent was waived. This study was examined and approved by THIN® Scientific Review Committee (protocol number 20 002 R1).

### Patient selection and study design

The target population enclosed all patients aged 18 years or older who were enroled in the THIN® UK database between 1994 and 2021. EHRs were eligible for inclusion if the corresponding patient had been enroled in the database for at least one year and had continuous follow-up with the same General Practitioner (GP) for at least one year. This criterion helped ensure consistency in medical records and minimise the risk of missing relevant health information. Importantly, the patients included in this analysis were unlikely to have had an unreported PDAC, as their regular consultations with the same GP allowed for continuous monitoring of their health status. We excluded patient records with inconsistent dates of birth or death, PDAC diagnosis before their inclusion in the database and those with non-adenocarcinoma pancreatic tumours.

Cases were identified from EHRs with a reported diagnosis of PDAC at any point during follow-up. To identify patient records with PDAC, we used the following THIN® UK specific codes instead of the ICD10 codes because they were more granular and specific regarding the pathology of the pancreatic tumour, as reported by the GP (Supplementary Table).

To create a control group, we randomly selected EHRs from the THIN® UK database without a reported cancer diagnosis at their last follow-up. Controls had to have at least one year of follow-up in the THIN® UK; this ensured that they consulted the same GP regularly, reducing the likelihood of having an unreported PDAC case among the control group. To ensure comparability between cases and controls and minimise potential confounding factors, we matched each case to 10 controls based on gender, age, follow-up duration, and year of inclusion, with the choice of 10 controls per case accounting for the prevalence of PDAC and in accordance with previous studies (Supplementary Fig.) [36, 37]. The follow-up period was defined as the time from inclusion in THIN® UK to either pancreatic cancer diagnosis (for cases) or the last follow-up visit (for controls).

### Candidate risk factors

For each patient consultation, the medical history, diagnosis for each visit, clinical data, blood test results, and prescriptions made by the primary care physicians were available. Based on a literature review and clinical relevance, we defined a comprehensive list of PDAC risk factors, including age, cigarette smoking, alcohol intake, medical comorbidities, clinical data, and laboratory test results [4, 19, 38, 39]. Relevant information was collected from the most recent medical record at least two years before the PDAC diagnosis for the cases, and at the last consultation for the controls (Supplementary Fig.). In the THIN® UK database, family history of PDAC was not systematically reported and was therefore excluded from our analysis.

### Comorbidities

The personal history of lung, head and neck, bladder cancers, melanoma as well as pancreatic diseases (including intraductal papillary mucinous neoplasm, acute and chronic pancreatitis, and pancreatic cysts), dyslipidemia, increased blood pressure, diabetes mellitus, cholecystitis, and *Helicobacter pylori* infection were collected from the EHRs. Missing variables were imputed as negative based on the assumption that patients who had consulted the same GP for more than one year were unlikely to present outdated records. Information on diabetes mellitus, changes of antidiabetic medication, dyslipidemia and increase blood pressure was verified through medical reimbursement records.

Information regarding alcohol intake and cigarette smoking were also obtained. Alcohol intake was either reported directly by the primary care physicians or imputed in patients with alcohol-related complications such as alcohol-induced cirrhosis. Given that the estimation of alcohol intake was not possible, it was defined as any alcohol use and alcohol use disorder including those with alcohol-related complications. Similarly, cigarette smoking was either mentioned in the medical record or assumed from the prescription of nicotine substitutes; it was classified as never or ever smoking.

### Clinical data

The anthropometric measures were collected directly from the EHRs at the last consultation with available corresponding data. Weight loss was defined as a decrease of 5% or more in body weight. Data on fatigue and abdominal pain were obtained whenever reported by the primary care physicians. These variables were collected from the most recent available time point at least two years before diagnosis among patient records with PDAC.

### Laboratory test results

Laboratory test results performed at least two years before the diagnosis PDAC for cases and censoring for controls were retrieved and considered as normal, abnormal (elevated or decreased) or missing. Only biologically plausible laboratory test results were included, specifically those related to liver function (alanine aminotransferase, aspartate aminotransferase, alkaline phosphatase, gamma-glutamyl transferase, bilirubin), pancreatic function (lipase, amylase), inflammatory state (complete blood count, erythrocyte sedimentation rate, C-reactive protein, lactate dehydrogenase), and cachexia (albumin, total cholesterol, glycated haemoglobin A1c).

### Statistical analysis

Patient characteristics, comorbidities and blood results were described using frequencies and proportions for categorical variables. We evaluated the association of various health conditions and test results with PDAC using conditional logistic regression. For the multivariable analysis, we employed backward selection with a significance threshold of *p* < 0.05 to identify the most relevant variables, computing odds ratios (OR) and their corresponding 95% confidence intervals (95% CI). All statistical analyses were performed using R 4.3.1 with the 'survival' package.

## Results

**Fig. 1:**
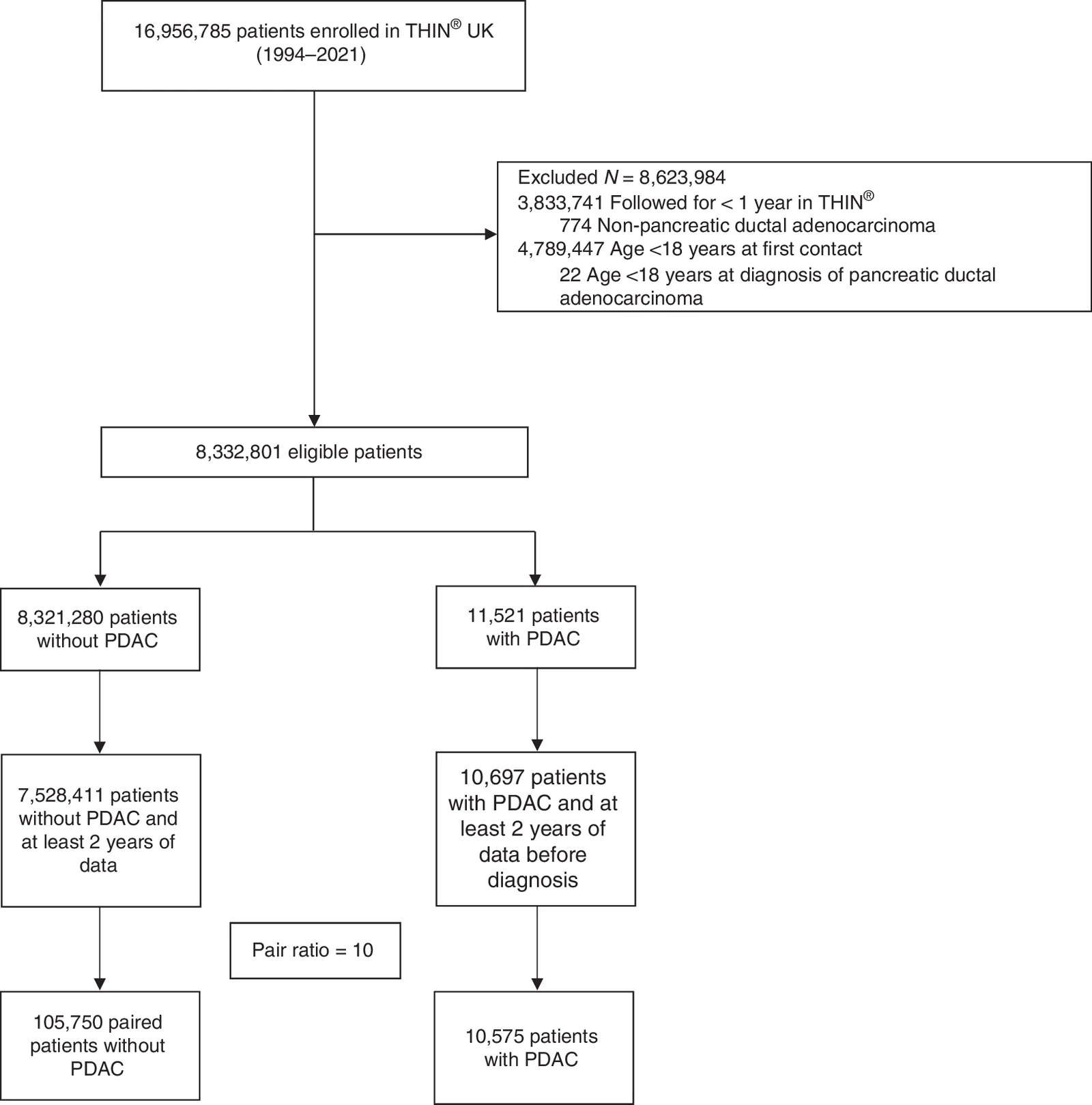
Participant flow diagram. PDAC: pancreatic ductal adenocarcinoma.

Our eligible matched cohort included 10,575 EHRs with PDAC and 105,750 controls (Fig. 1). The patient characteristics and comorbidities of cases and their matched controls are presented in Table 1. Males comprised 51.5% of the cohort and age over 70 years represented 52.2% of eligible EHR. Alcohol intake was more prevalent among cases (64.9%) compared to controls (62.1%). Additionally, a higher percentage of cases had an alcohol use disorder (5.6%) relative to controls (4.5%). Cigarette smoking was also more common in cases, with 56.6% having a history of smoking compared to 50.0% in controls.

**Table 1:** Characteristics of the study population at least two years before the diagnosis of PDAC (cases) or last visit (controls).

Variables of interest		Cohort with PDAC during follow up (total 10,575)	Cohort without PDAC during follow up (total 105,750)
Person-years follow up	Median	18	16
	Range	2–91	2–75
Gender	Male	5441 (51.5%)	54,410 (51.5%)
	Female	5134 (48.5%)	51,340 (48.5%)
	Missing	0	0
Age (years)	≤40	73 (0.7%)	730 (0.7%)
	41–50	432 (4.1%)	4320 (4.1%)
	51–60	1494 (14.1%)	14,940 (14.1%)
	61–70	3055 (28.9%)	30,550 (28.9%)
	>70	5521 (52.2%)	55,210 (52.2%)
	Missing	0	0
Alcohol intake	Alcohol use	6859 (64.9%)	65,674 (62.1%)
	Alcohol use disorder	588 (5.6%)	4726 (4.5%)
	No	1148 (10.9%)	11,586 (11%)
	Missing	2568 (24.3%)	28,490 (26.9%)
Cigarette smoking	Ever	5982 (56.6%)	52,835 (50%)
	Never	3741 (35.4%)	40,819 (38.6%)
	Not available	852 (24.3%)	12,096 (11.4%)
Comorbidities	Dyslipidemia	5108 (48.3%)	42,078 (39.8%)
	Increased blood pressure	4343 (41.1%)	39,718 (37.6%)
	Lung cancer	23 (0.2%)	647 (0.6%)
	Bladder cancer	73 (0.7%)	624 (0.6%)
	Head and neck cancer	17 (0.2%)	112 (0.1%)
	Melanoma	168 (1.6%)	1615 (1.5%)
	Pancreatic cysts	17 (0.2%)	27 ( < 0.1%)
	Pancreatitis	174 (1.6%)	683 (0.6%)
	Cholecystitis	158 (1.5%)	1145 (1.1%)
	Helicobacter pylori infection	46 (0.4%)	351 (0.3%)
	Diabetis mellitus ≤4 years	592 (5.6%)	5143 (4.9%)
	Diabetis mellitus >4 years	1152 (10.9%)	9721 (9.2%)
	Introduction or changes of antidiabetic medication over the last four years	1061 (10.0%)	7422 (7.0%)
**Clinical examination**			
Weight loss >5% within the previous year		206 (1.9%)	1742 (1.6%)
Fatigue		593 (5.6%)	6038 (5.7%)
Abdominal pain		1281 (12.1%)	11,684 (11%)
**Laboratory test results**			
Alanine aminotransferase	Missing	4936 (46.7%)	48,309 (45.7%)
	Elevated	2032 (19.2%)	12,525 (11.8%)
	Normal	3607 (34.1%)	44,916 (42.5%)
Aspartate aminotransferase	Missing	8056 (76.2%)	84,103 (79.5%)
	Elevated	579 (5.5%)	5277 (5%)
	Normal	1940 (18.3%)	16,370 (15.5%)
Alkaline phosphatase	Missing	3775 (35.7%)	40,604 (38.4%)
	Elevated	2566 (24.3%)	9361 (8.9%)
	Normal	4234 (40%)	55,785 (52.8%)
Gamma-glutamyl transferase	Missing	7590 (71.8%)	77,475 (73.3%)
	Elevated	1002 (9.5%)	9081 (8.6%)
	Normal	1983 (18.8%)	19,194 (18.2%)
Bilirubin	Missing	3589 (33.9%)	40,414 (38.2%)
	Elevated	1024 (9.7%)	3643 (3.4%)
	Normal	5962 (56.4%)	61,693 (58.3%)
Amylase	Missing	10,165 (96.1%)	102,847 (97.3%)
	Elevated	59 (0.6%)	287 (0.3%)
	Normal	351 (3.3%)	2616 (2.5%)
Erythrocyte sedimentation rate	Missing	6649 (62.9%)	70,639 (66.8%)
	Elevated	1344 (12.7%)	8811 (8.33%)
	Normal	2582 (24.4%)	26,300 (24.9%)
C-reactive protein	Missing	8293 (78.4%)	86,227 (81.5%)
	Elevated	1478 (14%)	9392 (8.9%)
	Normal	804 (7.6%)	10,131 (9.6%)
Lactate dehydrogenase	Missing	10,370 (98.1%)	104,059 (98.4%)
	Elevated	54 (0.5%)	414 (0.4%)
	Normal	151 (1.4%)	1277 (1.2%)
Albumin	Missing	3437 (32.5%)	40,961 (38.7%)
	Decreased	3200 (30.3%)	20,871 (19.7%)
	Normal	3938 (37.2%)	43,918 (41.5%)
Total cholesterol	Missing	3372 (31.9%)	38,427 (36.3%)
	Elevated	3128 (29.6%)	33,080 (31.3%)
	Normal	4075 (38.5%)	34,243 (32.4%)
Glycated haemoglobin A1c	Missing	8308 (78.6%)	85,108 (80.5%)
	Elevated	773 (7.3%)	5105 (4.8%)
	Normal	1494 (14.1%)	15,537 (14.7%)

*PDAC* pancreatic ductal adenocarcinoma.

Regarding comorbidities, dyslipidemia (48.3 vs. 39.8%) and increased blood pressure (41.1 vs. 37.6%) were more frequent in cases than in controls. Cases also had a slightly higher incidence of other conditions like pancreatitis (1.6 vs. 0.6%) and cholecystitis (1.5 vs. 1.1%). A notable difference was observed in the prevalence of diabetes; diabetes diagnosed for more than 4 years was recorded in 10.9% of cases versus 9.2% of controls, while diabetes diagnosed within 4 years was also more frequent among cases (5.6 vs. 4.9%).

We evaluated the association between the collected candidate risk factors and the occurrence of PDAC (Table 2). Univariable analyses suggested that pancreatic cysts (OR = 6.34, 95% CI 3.45–11.64), pancreatitis (OR = 2.58, 95% CI 2.18–3.05), dyslipidemia (OR = 1.53, 95% CI 1.46–1.60), cholecystitis (OR = 1.39, 95% CI 1.17–1.64), and increased blood pressure (OR = 1.18, 95% CI 1.13–1.23) were the clinical variables associated with an increased risk of PDAC. Elevated levels of total bilirubin (OR = 2.91, 95% CI 2.70–3.14), alanine aminotransferase (OR = 2.07, 95% CI 1.95–2.19), C-reactive protein (OR = 2.00, 95% CI 1.82–2.18), glycated haemoglobin A1c (OR = 1.57, 95% CI 1.43–1.72) and decreased levels of albumin (OR = 1.72, 95% CI 1.64–1.82) compared to normal values were the blood test abnormalities with the higher association with PDAC. Conversely, some markers and conditions with missing values were associated with lower odds of PDAC diagnosis.

**Table 2:** Uni and multivariable analysis of the risk factors for the occurrence of pancreatic ductal adenocarcinoma.

	Univariable analysis		Multivariable analysis	
Variables	OR (95% CI)	*p*-value	OR (95% CI)	*p*-value
Alcohol intake				
No	1.00		1.00	
Alcohol use	1.07 (1.00–1.14)	0.059	**1.09 (1.02**–**1.17)**	**0.011**
Missing	**0.88 (0.81**–**0.95)**	**0.0009**	1.06 (0.97–1.15)	0.170
Cigarette Smoking				
Never	1.00		1.00	
Ever	**1.25 (1.20**–**1.30)**	**<0.0001**	**1.19 (1.13**–**1.24)**	**<0.0001**
Missing	**0.72 (0.66-0.78)**	**<0.0001**	**0.75 (0.68**–**0.82)**	**<0.0001**
**Comorbidities**				
Dyslipidemia				
No	1.00		1.00	
Yes	**1.53 (1.46**–**1.60)**	**<0.0001**	**1.50 (1.43**–**1.57)**	**<0.0001**
Increased blood pressure				
No	1.00			
Yes	**1.18 (1.13**–**1.23)**	**<0.0001**		
Lung cancer				
No	1.00		1.00	
Yes	**0.35 (0.23**–**0.54)**	**<0.0001**	**0.29 (0.19**–**0.45)**	**<0.0001**
Bladder cancer				
No	1.00			
Yes	1.17 (0.92–1.50)	0.202		
Head and neck cancer				
No	1.00			
Yes	1.52 (0.91–2.53)	0.109		
Melanoma				
No	1.00			
Yes	1.04 (0.89–1.22)	0.623		
Pancreatic cyst				
No	1.00		1.00	
Yes	**6.34 (3.45**–**11.64)**	**<0.0001**	**4.39 (2.28**–**8.45)**	**<0.0001**
Pancreatitis				
No	1.00		1.00	
Yes	**2.58 (2.18**–**3.05)**	**<0.0001**	**2.16 (1.81**–**2.58)**	**<0.0001**
Cholecystitis				
No	1.00			
Yes	**1.39 (1.17**–**1.64)**	**0.0001**		
Helicobacter pylori infection				
No	1.00			
Yes	1.31 (0.96–1.79)	0.084		
Diabetis mellitus				
No	1.00			
Diagnosis ≤4 years	**1.19 (1.09**–**1.30)**	**0.0009**		
Diagnosis >4 years	**1.23 (1.15**–**1.31)**	**<0.0001**		
Introduction or changes of antidiabetic medication over the last four years				
No	1.00		1.00	
Yes	**1.49 (1.39**–**1.59)**	**<0.0001**	**1.19 (1.11**–**1.28)**	**<0.0001**
**Clinical examination**				
Weight loss >5% within the previous year				
No	1.00			
Yes	1.00 (0.86–1.15)	0.975		
Missing	**0.74 (0.71**–**0.78)**	**<0.0001**		
Fatigue				
No	1.00			
Yes	**0.98 (0.90**–**1.07)**	0.664		
Abdominal pain				
No	1.00			
Yes	**1.11 (1.04**–**1.18)**	**0.0008**		
**Laboratory test results**				
Alanine aminotransferase				
Normal	1.00		1.00	
Elevated	**2.07 (1.95**–**2.19)**	**<0.0001**	**1.76 (1.66**–**1.87)**	**<0.0001**
Missing	**1.32 (1.26**–**1.39)**	**<0.0001**	**1.73 (1.63**–**1.84)**	**<0.0001**
Aspartate aminotransferase				
Normal	1.00			
Elevated	0.92 (0.84–1.02)	0.113		
Missing	**0.79 (0.75**–**0.84)**	**<0.0001**		
Alkaline phosphatase				
Normal	1.00		1.00	
Elevated	**3.70 (3.51**–**3.91)**	**<0.0001**	**3.21 (3.03**–**3.39)**	**<0.0001**
Missing	**1.25 (1.19**–**1.32)**	**<0.0001**	**1.62 (1.51**–**1.74)**	**<0.0001**
Gamma-glutamyl transferase				
Normal	1.00		1.00	
Elevated	1.07 (0.98–1.16)	0.111		
Missing	**0.94 (0.89**–**0.99)**	**0.023**		
Total bilirubin				
Normal	1.00		1.00	
Elevated	**2.91 (2.70**–**3.13)**	**<0.0001**	**2.48 (2.29**–**2.68)**	**<0.0001**
Missing	**0.84 (0.79**–**0.89)**	**<0.0001**	0.97 (0.90–1.04)	0.430
Amylase				
Normal	1.00			
Elevated	**1.53 (1.13**–**2.07)**	**0.006**		
Missing	**0.73 (0.65**–**0.82)**	**<0.0001**		
Erythrocyte sedimentation rate				
Normal	1.00		1.00	
Elevated	**1.56 (1.45**–**1.67)**	**<0.0001**	**1.27 (1.18**–**1.37)**	**<0.0001**
Missing	**0.94 (0.90**–**0.99)**	**0.021**	0.95 (0.90–1.00)	0.061
C-reactive protein				
Normal	1.00			
Elevated	**2.00 (1.82**–**2.18)**	**<0.0001**		
Missing	**1.19 (1.10**–**1.28)**	**<0.0001**		
Lactate dehydrogenase				
Normal	1.00			
Elevated	1.10 (0.79–1.53)	0.560		
Missing	**0.84 (0.71**–**1.00)**	**0.047**		
Albumin				
Normal	1.00		1.00	
Decreased	**1.72 (1.64**–**1.82)**	**<0.0001**	**1.54 (1.46**–**1.62)**	**<0.0001**
Missing	**0.83 (0.78**–**0.88)**	**<0.0001**	**0.81 (0.75**–**0.87)**	**<0.0001**
Total cholesterol				
Normal	1.00			
Elevated	0.78 (0.74–0.82)	**<0.0001**		
Missing	**0.66 (0.62**–**0.70)**	**<0.0001**		
Glycated haemoglobin A1c				
Normal	1.00			
Elevated	**1.57 (1.43**–**1.72)**	**<0.0001**		
Missing	1.00 (0.94–1.07)	0.985		

*OR* odds ratio, 95% *CI* confidence interval.

Statistically significant associations (*p* < 0.05) are shown in bold.

Multivariable analysis showed that pancreatic cysts (OR = 4.39, 95% CI 2.28–8.45) and elevated alkaline phosphatase (OR = 3.21, 95% CI 3.03–3.39) were the strongest independent variables associated with PDAC. Clinical risk factors independently associated with an increased risk of PDAC included pancreatitis (OR = 2.16, 95% CI 1.81–2.58), dyslipidemia (OR = 1.50, 95% CI 1.43–1.57), cigarette smoking (OR = 1.19, 95% CI 1.13–1.24), alcohol intake (OR = 1.09, 95% CI 1.02–1.17), and introduction or changes of antidiabetic medication over the last four years (OR = 1.19, 95% CI 1.11–1.28). Laboratory markers linked to higher PDAC risk included elevated alanine aminotransferase (OR = 1.76, 95% CI 1.66–1.87), total bilirubin (OR = 2.48, 95% CI 2.29–2.68), and erythrocyte sedimentation rate (OR = 1.27, 95% CI 1.18–1.37), as well as decreased albumin (OR = 1.54, 95% CI 1.46–1.62).

## Discussion

This large comprehensive nested case-control study provides insights into the risk factors associated with PDAC in the general population of UK as reported in the THIN® UK database. It stands out from many previous investigations by addressing the potential risk factors present at least two years before PDAC diagnosis as it highlights a potential window of opportunity for early intervention before the disease becomes clinically apparent. This study provides evidence that a more comprehensive and multifactorial approach to risk assessment may outperform traditional high-risk screening criteria, which primarily emphasise family history or genetic predisposition. By analysing comorbidities, clinical data, and blood test results at least two years before PDAC diagnosis, this study offers an interesting perspective of clinical significance with the early identification of individuals at increased risk of PDAC [4, 40].

This study showed a strong association between alcohol intake and increased PDAC risk, especially in cases involving alcohol use disorder. The higher prevalence of alcohol use and alcohol use disorders among cases aligns with prior research demonstrating a dose-dependent relationship between alcohol consumption and PDAC risk [41]. Cigarette smoking and alcohol intake are well-established risk factors for PDAC, the relatively modest association observed in this study suggests that other risk factors may play a more significant role in certain populations [42, 43]. However, reducing smoking and alcohol consumption remains a relevant preventive strategy due to their modifiable nature. The independent association between dyslipidemia and PDAC adds to the growing body of literature linking metabolic disorders and PDAC [19]. The underlying mechanisms may involve chronic inflammation, insulin resistance, and alterations in the tumour microenvironment [44, 45].

Another finding of this study is the strong association between liver function abnormalities and PDAC risk, even two years before diagnosis. Elevated alkaline phosphatase and bilirubin levels emerged as the strongest independent risk factors of PDAC in the multivariable analysis. This aligns with recent research suggesting that subtle changes in liver function may precede clinically apparent PDAC by several years [46, 47]. On the other hand, missing bilirubin data was associated with lower odds of PDAC, reflecting the potential for diagnostic gaps in patients without available blood test results. The study also observed a significant relationship between elevated erythrocyte sedimentation rate and PDAC risk. Although erythrocyte sedimentation rate is a non-specific marker of inflammation, its elevation may reflect underlying chronic inflammatory states that promote pancreatic carcinogenesis [45]. This finding suggests that integrating inflammatory markers into risk prediction models for PDAC could enhance early detection.

The study’s results regarding diabetes mellitus favour the bidirectional relationship between diabetes and PDAC, which was substantiated by the higher prevalence of long-standing and recent-onset diabetes among records of cases [48]. While long-standing diabetes is a known risk factor for PDAC, recent-onset diabetes may, in some cases, be an early manifestation of the disease itself [49]. This highlights the importance of careful monitoring of new-onset diabetes in older adults, particularly when accompanied by other risk factors identified in this study. Last, the observed associations between PDAC and other pancreatic comorbidities, such as pancreatic cysts and pancreatitis are consistent with previous research [50, 51]. These conditions may create a pro-inflammatory environment in the pancreas that promotes carcinogenesis. However, the relatively low prevalence of these conditions in the study population suggests that they may have limited utility as standalone risk factors for population-wide screening. Interestingly, obesity was not associated with an increased risk in the medium term although it has long been described as a major risk factor for pancreatic cancer. This could be due to a lack of medium term effect, or to the effect of certain drugs these patients may receive, including GLP1 inhibitors [52, 53].

The main strength of this study is its use of EHR with a long longitudinal follow-up to capture a wide range of potential risk factors, including laboratory parameters that may not be routinely included in traditional epidemiological studies [54]. It provides valuable insights but several limitations should be acknowledged. First, the retrospective nature of the analysis and reliance on EHR may introduce bias due to incomplete or inconsistent data recording. It also highlights the challenges of missing data in real-world clinical settings, as evidenced by the association between missing laboratory results and PDAC risk. Second, the study’s focus on a UK population may limit generalisability to other populations with different genetic backgrounds, lifestyle factors or healthcare systems. Third, while the study identifies several potentially modifiable risk factors, such as alcohol intake and dyslipidemia, the impact of interventions targeting these factors on PDAC risk reduction remains to be determined.

## Conclusion

This comprehensive analysis of risk factors for PDAC in the general population showed that primary care EHR can identify individuals at medium-term increased risk of PDAC using widely accessible data in daily practice, which is particularly important for clinical translation. The identified risk factors can eventually provide practical guidance to increase the vigilance of physicians in patients where multiple risk indicators converge. They can be insightful for developing PDAC screening and early detection strategies, mainly in regards to the identification of subtle changes in biomarkers that may precede clinical diagnosis of PDAC by several years. Current guidelines, which restrict screening to high-risk individuals based on family history and rare genetic variants, may be missing opportunities for early detection in the general population [23]. The constellation of risk factors identified in this study suggests that a more nuanced approach to risk stratification could improve the yield of screening efforts and lays the groundwork for developing prediction models. However, translating these findings into clinical practice will require careful consideration. The relatively low incidence of PDAC in the general population means that even with improved risk prediction models, the positive predictive value of any screening approach is likely to be limited. This underscores the need for specific screening modalities to minimise false positives and increase the prediction accuracy.

## Supplementary information


Supplementary Table
Supplementary Figure Legend
Supplementary Figure


## Data Availability

THIN® Database (A Cegedim Proprietary Database) analysed for this paper is the private property of CEGEDIM. Access to the data can be granted upon reasonable written request and approval by the THIN® Scientific Review Committee.
