# Correction: Identifying health conditions associated with an increased risk of pancreatic ductal adenocarcinoma at medium term in nationwide electronic health records of primary care physicians

**DOI:** 10.1038/s41416-026-03597-6

**Published:** 2026-08-31

**Authors:** Elie Rassy, Suzette Delaloge, Yannis Slaouti, Thomas Pudlarz, Béranger Lekens, Alice Boilève, Stefan Michiels, Maryam Karimi

**Affiliations:** 1 Department of Cancer Medicine, Gustave Roussy, University Paris-Saclay, Villejuif, France; 2 Department of Biostatistics and Epidemiology, Gustave Roussy, University Paris-Saclay, Villejuif, France; 3 Oncostat U1018, Inserm, University Paris-Saclay, labeled Ligue Contre le Cancer, Villejuif, France; 4 CEGEDIM R&D Boulogne-Billancourt, Paris, France; 5 Université Paris Saclay, Orsay, France; 6 Gustave Roussy, INSERM U1279, Villejuif, France

**Keywords:** Cancer epidemiology, Gastrointestinal cancer

Correction to: *British Journal of Cancer* https://doi.org/10.1038/s41416-025-03172-5, published online 30 August 2025

In this article, the author’s name Maryam Karimi was incorrectly written as Mariam Karimi.

The original article has been corrected.

