# Supplementary material for: Identifying health conditions associated with an increased risk of pancreatic ductal adenocarcinoma at medium term in nationwide electronic health records of primary care physicians
Source: Br J Cancer. 2025 Aug 30;133(9):1317–25. doi: 10.1038/s41416-025-03172-5 (PMC12572402; doi:10.1038/s41416-025-03172-5)
Supplement: Supplementary file 1 — Supplementary Table [file 41416_2025_3172_MOESM1_ESM.docx]

| Supplementary Table : The codes used to define pancreatic ductal adenocarcinoma | | |
| --- | --- | --- |
| ICD-10 | THIN® UK Code | Label |
| C25.0 | INUK.B170.00 | Malignant neoplasm of head of pancreas |
| C25.1 | INUK.B171.00 | Malignant neoplasm of body of pancreas |
| C25.2 | INUK.B172.00 | Malignant neoplasm of tail of pancreas |
| C25.3 | INUK.B173.00 | Malignant neoplasm of pancreatic duct |
| C25.7 | INUK.B17y.00 | Malignant neoplasm of other specified sites of pancreas |
| C25.7 | INUK.B17yz00 | Malignant neoplasm of specified site of pancreas NOS |
| C25.9 | INUK.B17..00 | Malignant neoplasm of pancreas |
| C25.9 | INUK.B17z.00 | Malignant neoplasm of pancreas NOS |
| ICD: international classification of disease | | |
