# Supplementary material for: Identifying health conditions associated with an increased risk of pancreatic ductal adenocarcinoma at medium term in nationwide electronic health records of primary care physicians
Source: Br J Cancer. 2025 Aug 30;133(9):1317–25. doi: 10.1038/s41416-025-03172-5 (PMC12572402; doi:10.1038/s41416-025-03172-5)
Supplement: Supplementary file 2 — Supplementary Figure Legend [file 41416_2025_3172_MOESM2_ESM.docx]

Supplementary figure title : Schema of the study design
